# Prevalence and factors associated with intestinal schistosomiasis and human fascioliasis among school children in Amhara Regional State, Ethiopia

**DOI:** 10.1186/s41182-021-00326-y

**Published:** 2021-05-11

**Authors:** Teshome Bekana, Nega Berhe, Tadesse Eguale, Mulugeta Aemero, Girmay Medhin, Begna Tulu, Yirgalem G/hiwot, Song Liang, Wei Hu, Berhanu Erko

**Affiliations:** 1 Aklilu Lemma Institute of Pathobiology, Addis Ababa University, Addis Ababa, Ethiopia; 2 Department of Biomedical Sciences, Faculty of Public Health and Medical Science, Mettu University, Mettu, Ethiopia; 3 Department of Medical Parasitology, College of Medicine & Health Sciences, University of Gondar, P. O. Box 196, Gondar, Ethiopia; 4 Department of Medical Laboratory Sciences, Bahir Dar University, Bahir Dar, Ethiopia; 5 Department of Environmental and Global Health, College of Public Health and Health Professions, and Emerging Pathogens Institute, University of Florida, Gainesville, FL 32610 USA; 6 Department of Microbiology and Microbial Engineering, School of Life Science, Fudan University, Shanghai, China

**Keywords:** Prevalence, Risk factors, *S. mansoni*, *Fasciola* species, Amhara Regional State, Ethiopia

## Abstract

**Background:**

Schistosomiasis and fascioliasis are digenean parasitic infections and are among the neglected tropical diseases that have both medical and veterinary importance. They are found mainly in areas having limited access to safe water supply and improved sanitation.

**Methods:**

A cross-sectional study was conducted to determine the prevalence of *Schistosoma mansoni* and *Fasciola* species infections and to identify associated risk factors among school children in Amhara Regional State, Ethiopia. Stool specimens were collected from 798 children (419 males, 379 females) and processed using Kato-Katz and formol-ether concentration techniques. A semi-structured questionnaire was used to collect socio-demographic and other exposure information to explore potential risk factors for the infections.

**Results:**

The overall prevalence of *S. mansoni* and *Fasciola* species infections was 25.6% (95% confidence interval (CI): 22.5-28.6) and 5.5% (95% CI: 3.9-7.1), respectively. *S. mansoni* was present in all surveyed schools with the prevalence ranging from 12.8% (16/125; 95% CI = 5.6-20.0) to 39.7% (64/161; 95% CI = 32.2-47.2) while *Fasciola* species was identified in five schools with the prevalence ranging from 2.5% (4/160; 95% CI = 0.001–4.9) to 9.8% (13/133; 95% CI = 4.7–14.8). The prevalence of *S. mansoni* infection was significantly associated with swimming in rivers (Adjusted odds ratio (AOR): 1.79, 95% CI, 1.22–2.62; *P*=0.003), bathing in open freshwater bodies (AOR, 2.02; 95% CI, 1.39–2.94; *P*<0.001) and engaging in irrigation activities (AOR, 1.69; 95% CI, 1.19-2.39; *P*=0.004), and was higher in children attending Addis Mender (AOR, 2.56; 95% CI, 1.20–5.46; *P*=0.015 ) and Harbu schools (AOR, 3.53; 95% CI, 1.64–7.59; *P*=0.001). *Fasciola* species infection was significantly associated with consumption of raw vegetables (AOR, 2.47; 95% CI, 1.23-4.97; *P*=0.011) and drinking water from unimproved sources (AOR, 2.28; 95% CI, 1.11–4.70; *P*=0.026).

**Conclusion:**

Both intestinal schistosomiasis and human fascioliasis are prevalent in the study area, affecting school children. Behaviors and access to unimproved water and sanitation are among significant risk factors. The findings are instrumental for targeted interventions.

## Background

Neglected tropical diseases (NTDs) pose a substantial threat to global health, affecting billions of population and causing more than 25 million disability-adjusted life years (DALYs) [1, 2]. Diseases caused by digenean parasitic trematodes, including the blood flukes *Schistosoma mansoni*, *S. haematobium*, *S. japonicum*, *S. mekongi*, *S. guineensis*, and *S. intercalatum*, and liver flukes *Fasciola hepatica* and *F. gigantica*, are among the neglected tropical diseases in the tropics and subtropics regions [3]. Worldwide, schistosomiasis and fascioliasis infect more than 200 million and 2.4 million people, respectively, with 850 million people being at the risk of both infections [3]. These infections typically occur in areas of low- and middle-income countries (LMICs) with lack of improved water supply and adequate sanitation [1] and their transmission is linked to environmental factors, such as temperature, rainfall, and water bodies, which can affect the distribution of intermediate snail hosts and alter the development rate of the parasites [1, 2].

Schistosomiasis is a water-based parasitic disease caused by trematode worms of the genus *Schistosoma* and transmitted via contact with freshwater bodies contaminated by cercariae [3]. In Ethiopia, there are two schistosome species known to infect humans: *S. mansoni* and *S. haematobium*. The former, causing intestinal schistosomiasis, is widely distributed across many areas of the country, whereas the latter, causing urogenital schistosomiasis is restricted to lower altitude areas of the country [4]. It is estimated that more than 5 million people are infected with schistosomes and about 37.5 million, particularly those living in irrigated agricultural areas, are at risk of infection in Ethiopia [5]. Previous studies conducted in several regions of the country revealed a correlation between schistosome transmission and recent environmental changes resulting from extensive water resource developments [4, 5]. In addition, anthropogenic changes such as increased population growth and movements, altered agricultural practices, lack of adequate drinking water, and poor living conditions have played an important role in spreading schistosomiasis throughout the country [4, 6].

Fascioliasis is a food- or water-borne trematodiasis that can be transmitted via consumption of uncooked or unwashed aquatic vegetables or through drinking water contaminated with infective metacercariae cysts [7]. In the past, public health impact associated with *Fasciola* species infection remains less well understood, in part due to lack of data on the parasites’ distribution and their infection status in humans [8]. However, recent studies conducted in many countries show that the infection has a vast geographic distribution throughout Europe, South America, and Oceania, where only *F. hepatica* infection commonly occurs, and in Africa and Asia, where the infection is caused by either *F. hepatica* or *F*. *gigantica* [7, 8]. Owing to its widespread distribution, the World Health Organization(WHO) recognized human fascioliasis as one of the most important foodborne parasitic diseases and included in the list of neglected tropical diseases in 2010 [9].

Although schistosomiasis is a widely reported parasitic disease in many areas of the country [4, 5], information on prevalence, intensity, and determinants of *S. mansoni* infection in the present study area are lacking. In addition, although bovine fascioliasis is highly prevalent and widely distributed, causing considerable economic loss to the livestock production in Ethiopia [10], only limited studies reported on human fascioliasis [10–12]. Data on human infections by these parasites in understudied but suspected regions are needed to inform targeted surveillance and/or intervention. Therefore, this study was initiated to assess the prevalence and factors associated with *S. mansoni* and *Fasciola* species infections among school children in selected areas of Amhara Regional State, Ethiopia.

## Methods

### Study setting

**Fig. 1:**
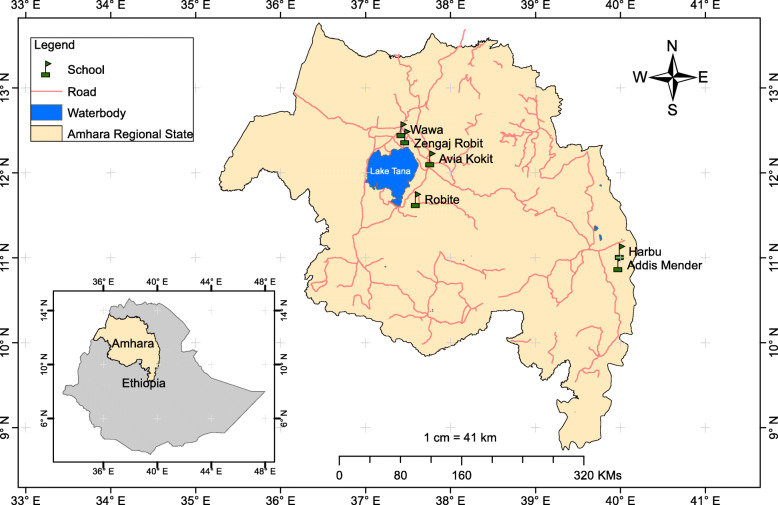
Map of study area

A cross-sectional survey was conducted between December 2017 and February 2018 in six primary schools in three districts—Kalu (South Wello), Fogera (South Gondar administrative zone), and Dembia (Central Gondar administrative zone)—Amhara Regional States, Ethiopia (Fig. 1). The area has tropical climatic conditions, characterized by a short rainy season from March to May, a long rainy season from June to September, and a dry season from November to February. The area has an elevation of 1484-2100 meters above sea level with a mean annual rainfall ranging from 750 to 1500 mm and a mean temperature ranging from 10 °C to 30 °C. People living in the area earn their livelihood through producing crops (e.g., rice, bean, and maize) and ranching livestock, mainly cattle, sheep, and goats. The main vegetables grown and consumed in this area are cabbage cauliflower, green chili, cherry tomatoes, and capsicum annum, which are often eaten raw or washed using water from unimproved water sources. Inhabitants have limited access to improved sanitation facilities [13]. The area has many permanent water collections, swampy areas, and rivers which contain the intermediate host snails such as *Biomphalaria pfeifferi*, *Lymnaea truncatula*, and *L.natalenesis* [14, 15].

### Study design and sample size

A school-based cross-sectional survey was conducted to assess the prevalence and factors associated with *S. mansoni* and *Fasciola* species infections among school children in six primary schools (Addis Mender, Harbu, Avia Kokit, Robite, Zengaj Robit, and Wawa) of Amhara Regional State. Sample size was computed using statistical formula $n=\frac{Z^2\left(1-\frac{\alpha }{2}\right)P\left(1-P\right)}{d^2}$ [16] where, *n* is sample size, *Z* is standard score for level of confidence, *P* is expected prevalence, and *d* is margin of error. By taking prevalence of 49% for *S. mansoni* infection from the previous study [17], 95% confidence level and 5% margin of error, the calculated sample size became 384. Considering a design effect of 2 and non-response rate of 10%, the final sample size was estimated to be 845.

### Sampling technique

The study area was selected based on presence of favorable environment for the breeding of snail hosts, practice of small-scale irrigations, suspected high burden of schistosomiasis (based on the 2013/2014 national mapping of NTDs) [18], and endemicity for animal fascioliasis. Class roster containing names, age, and sex of the children attending grades 1-4 were obtained from the head teachers of all schools in order to get the number of children aged 6 to 15 years. Thereafter, a total of 845 school children (the desired sample size) were proportionally allocated to the six schools based on the number of eligible children in each school. Within each school, a systematic sampling technique was used to select the study participants using class registers as the sampling frame. Of the eligible children, 36 did not provide any stool specimens and 9 provided insufficient stool specimens—were excluded from the study. This resulted in 798 children who were included in final analysis.

### Questionnaire survey

A pre-tested questionnaire assessing risks for *S. mansoni* and *Fasciola* species infections was administered to the childrens’ parents and/or guardians in the household setting, while the participating children were interviewed at their schools. The parents (mothers or head of the household) were interviewed to collect information on socio-demographic characteristics, source of drinking water, children’s habit of consuming vegetables, irrigation practices, and ownership of domestic animals. In addition, they were asked if their children had consumed raw liver 2 weeks prior to conducting this study to rule out the occurrences of false positive fascioliasis. Children were asked about their water contact habits, such as washing clothes, fetching water, water body contact while crossing, swimming, bathing in open water bodies, and whether they engaged in irrigation farming while assisting their parents. Trained study team members and school teachers conducted the interview using the local langue (Amharic).

### Specimen collection and processing

Children aged 6 to 15 years who obtained written informed consent from their parents/guardians were given clean plastic sheet with wooden applicator stick and asked to provide their own feces (approximately 5 g). Double Kato-Katz thick smears (using 41.7 mg template) were prepared from each specimen within 3 h of specimen collection in the study area. The slides were examined after 24 h of preparation by experienced laboratory technicians. The remaining portion of specimen (approximately 3 g) was preserved in 10% formalin solution before being processed using the formol-ether concentration technique [19]. The processed specimens were then examined within 2 h of sample preparation at the Medical Parasitology Laboratory of the Aklilu Lemma Institute of Pathobiology, Addis Ababa University. The formol–ether concentration technique was used for detection of *S*. *mansoni* and *Fasciola* species eggs, and the Kato-Katz technique was used for detection and quantification of eggs of both parasites. To estimate infection intensity, the number of eggs counted in both Kato–Katz slides was converted into eggs per gram of stool (epg) by multiplying the mean of eggs counted in both slides by a factor of 24 [19, 20]. Infection intensity of *S. mansoni* was classified as light (1–99 epg), moderate (100–399 epg), and heavy (> 400 epg), based on the WHO guideline [20]. The detection of *S*. *mansoni* and *Fasciola* species eggs by either Kato-Katz or formol–ether concentration technique was considered positive and the absence of at least 1 egg for each parasite was recorded as negative. To ensure consistency in egg counting, 10% of the Kato-Katz smears were re-examined by a senior laboratory technician.

### Data analysis

Data were computerized in Microsoft Excel version 2007 (MS Corporation, Washington, USA) and analyzed using the Statistical Package for Social Scientists (SPSS) version 20. (SPSS Inc., Chicago, IL, USA). Prevalences of *S. mansoni* and *Fasciola* species infections were determined by using descriptive statistical analysis. Student’s *t* test was used to test any statistical difference in infection intensity for *S. mansoni* and *Fasciola* species infections between sex and age groups. Univariate and multivariate logistic regression analysis were used to identify factors associated with the infections. Any association with a *P* values less than 5% was considered statistically significant.

## Results

### Characteristics of the study participants

A total of 845 school children selected from six primary schools were invited to participate in the study. Among these, 798 (94.4%) provided sufficient stool specimens and complete information. The mean age of the children was 10.6 year (standard deviation=2.1 years) with a range of 6-15 years, and 419 (52.5%) of the children were male. Majority of the children (62.2%) drink water from unimproved supply, 57.1% had the history of uncooked vegetable consumption, and 45.4% were involved in irrigational activities. The families of most children (96.4%,) owned domestic animals, including cattle, sheep, and goats.

### Prevalence of *S*. *mansoni* and *Fasciola* species infections

Out of 798 children examined, 403(50.5%) had one or more intestinal helminths. Two hundred four (25.6%) of the children were infected with *S*. *mansoni*, while 44 (5.5%) were infected with *Fasciola* species (Tables 1 and 2). In total, 1.4% of school children (*n*=11) were infected with both *S. mansoni* and *Fasciola* species. A significant negative association was observed between the two infections (correlation coefficient =−0.03). Other intestinal helminth parasites identified were *Ascaris lumbricoides* (13.0%), hookworms (8.3%), *Trichuris trichiura* (6.2%), *Hymenolepis nana* (2.0%), *Enterobius vermicularis* (0.9%), and *Taenia* species (0.5%). In relation to age, the highest prevalence of *S. mansoni* infection was observed in the 9-11 years age group (29.2%), followed by 12-15 years (23.6%), and 6-8 years (20.6%). The prevalence of *S. mansoni* infection varied considerably across the six schools, the highest being in Addis Mender School (64/161, 39.7%) while the least was in Avia Kokit School (16/125, 12.8%) (Table 1). The prevalence of *Fasciola* species infection in the children also varied considerably across schools, with the highest in Zengaj Robit (13/133, 9.8%), followed by Avia Kokit (11/125, 8.8%), Robite (12/156, 7.7%), Wawa (4/63, 6.3%), Harbu (4/160, 2.5%), and the least in Addis Mender where no infection was found (0) (Table 2).

**Table 1:** Prevalence of *S.mansoni* infection among the school children in Amhara Regional State, Ethiopia

Variable	Number examined	Prevalence^a^ (95% CI)	Univariate COR (95% CI)	*P* value	Multivariate AOR (95% CI)	*P* value
**Overall**	798	25.6 (22.5-28.6)				
**Age (years)**.						
6-8	184	20.6 (14.8-26.4)	1			
9-11	377	29.2 (24.6-33.8)	0.84 (0.53-1.34)	0.467	0.94 (0.57-1.56)	0.816
12-15	237	23.6 (18.2-29.0)	1.33 (0.92-1.93)	0.132	1.29 (0.86-1.94)	0.212
**Gender**						
Male	419	28.4 (23.9-32.7)	0.73 (0.53-1.01)	0.054	0.82 (0.58-1.15)	0.250
Female	379	22.4 (18.2-26.6)	1			
**Uncooked vegetable consumption**						
Yes	456	25.9 (21.9-29.9)	1.04 (0.75-1.43)	0.825		
No	342	25.1 (20.5-29.7)	1			
**Source of drinking water**						
Unimproved	496	27.0 (23.1-30.9)	1.23 (0.88-1.71)	0.229		
**Improved**	302	23.2 (18.4-27.9)	1			
**Washing clothes**						
Yes	350	29.1 (24.3-33.8)	1.39 (1.01-1.92)	0.041	1.31 (0.93-1.85)	0.125
No	448	22.8 (18.9-26.7)	1		1	
**Fetching water**						
Yes	385	29.6 (25.1-34.2)	1.51 (1.09-2.08)	0.012	1.32 (0.93-1.86)	0.117
No	413	21.8 (17.8-26.7)	1		1	
**Water body contact while crossing**						
Yes	162	24.1 (17.5-30.7)	0.91 (0.61-1.35)	0.626		
No	636	25.9 (22.5-29.3)	1			
**Swimming in rivers**						
Yes	372	29.6 (24.9-34.2)	1.48 (1.08-2.04)	0.016	1.79 (1.22-2.62)	0.003
No	426	22.1 (18.2-26.0)	1		1	
**Bathing in open freshwater bodies**						
Yes	290	30.7 (25.4-36.0)	1.51 (1.09-2.09)	0.012	2.02 (1.39-2.94)	<0.001
No	508	22.6 (18.9-26.2)	1		1	
**Participating in irrigation farming**						
Yes	362	30.4 (25.7-35.1)	1.59 (1.15-2.19)	0.005	1.69 (1.19-2.39)	0.004
No	436	21.6 (17.7-25.4)	1		1	
**Ownership domestic animals**						
Yes	769	26.0 (22.9-29.2)	2.19 (0.75-6.39)	0.149		
No	29	13.8 (12.5-26.3)	1			
**School**						
Addis Mender	161	39.7 (32.2-47.2)	3.12 (1.51-6.43)	0.002	2.56 (1.20-5.46)	0.015
Harbu	160	38.1 (30.6-45.6)	2.91 (1.41-6.01)	0.004	3.53 (1.64-7.59)	0.001
Avia Kokit	125	12.8 (5.6-20.0)	0.69 (0.30-1.60)	0.391	0.79 (0.33-1.91)	0.604
Robite	156	18.6 (12.5-24.7)	1.08 (0.50-2.32)	0.845	0.72 (0.32-1.61)	0.419
Zengaj Robit	133	17.3 (10.9-23.7)	0.99 (0.45-2.18)	0.977	0.84 (0.37-1.91)	0.672
Wawa	63	17.5 (8.1-26.9)	1		1	

Abbreviations: *AOR* Adjusted odds ratio, *CI* Confidence interval, *COR* Crude odds ratio

^a^Determined by both techniques

**Table 2:** Prevalence of *Fasciola* species infection among the school children in Amhara Regional State, Ethiopia

Variable	Number examined	Prevalence^a^ (%) (95% CI)	Univariate COR (95% CI)	*P* value	Multivariate AOR (95% CI)	*P* value
**Overall**	798	5.5 (3.9-7.1)				
**Age (years)**						
6-8	184	3.8 (1.1-6.6)	1			
9-11	377	6.1 (3.7-8.5)	0.63 (0.25-1.59)	0.329		
12-15	237	5.9 (2.9-8.9)	1.03 (0.52-2.05)	0.922		
**Gender**						
Male	419	5.7 (3.5-7.9)	0.92 (0.49-1.69)	0.781		
Female	379	5.3 (3.0-7.6)	1			
**Uncooked vegetable consumption**						
Yes	456	7.2 (4.8-9.6)	2.35 (1.17-4.72)	0.016	2.47 (1.23-4.97)	0.011
No	342	3.2 (1.3-5.1)	1		1	
**Source of drinking water**						
Unimproved	496	6.8 (4.6-9.0)	2.15 (1.05-4.41)	0.037	2.28 (1.11-4.70)	0.026
Improved	302	3.3 (1.3-5.3)	1		1	
**Washing clothes**						
Yes	350	6.0 (3.5-8.5)	1.18 (0.64-2.17)	0.595		
No	448	5.1 (3.1-7.1)	1			
**Fetching water**						
Yes	385	5.2 (2.9-7.4)	0.89 (0.48-1.64)	0.703		
No	413	5.8 (3.5-8.1)	1			
**Water body contact while crossing**						
Yes	162	7.4 (3.4-11.4)	1.51 (0.76-3.0)	0.240		
No	636	5.0 (3.3-6.7)	1			
**Swimming in rivers**						
Yes	372	4.8 (2.6-6.9)	0.78 (0.42-1.45)	0.436		
No	426	6.1 (3.8-8.4)	1			
**Bathing in open freshwater bodies**						
Yes	290	5.9 (3.2-8.6)	1.12 (0.59-2.07)	0.745		
No	508	5.3 (3.4-7.2)	1			
**Participating in irrigation farming**						
Yes	362	5.8 (3.4-8.2)	1.48 (0.80-2.72)	0.211		
No	436	5.3 (3.2-7.4)	1			
**Ownership of domestic animals**						
Yes	769	5.5 (3.9-7.1)	0.78 (0.18-3.39)	0.740		
No	29	6.9 (5.1-8.7)	1			
**School**						
Addis Mender	161	0				
Harbu	160	2.5 (0.001-4.9)	0.38 (0.09-1.56)	0.179		
Avia Kokit	125	8.8 (3.8-137)	1.42 (0.43-4.66)	0.560		
Robite	156	7.7 (3.5-11.9)	1.23 (0.38-3.97)	0.730		
Zengaj Robit	133	9.8 (4.7-14.8)	1.59 (0.49-5.11)	0.430		
Wawa	63	6.3 (0.3-12.3)	1			

Abbreviations: *AOR* Adjusted odds ratio, *CI* Confidence interval, *COR* Crude odds ratio

^a^Determined by both techniques

### Intensity of *S. mansoni* and *Fasciola* species infections by age and gender

Intensities of *S. mansoni* and *Fasciola* species infections stratified by age and gender are summarized in Table 3. The mean intensity (i.e., geometric mean) of *S. mansoni* infection was 104.3 epg (95% CI, 63.5-145.1) and ranged from 24 eggs to 1944 epg. Among *S. mansoni* infected children, heavy intensity was detected in 11.5% (20/174), whereas moderate and light infection intensities were found in 33.3% (58/174) and 55.2% (96/174) of the children, respectively. The male had higher infection intensity than female (*P*=0.028). The overall mean of *Fasciola* species egg counts for the infected children was 93.7 epg (95% CI, 36.5-150.9), with absolute egg counts ranging from 24 to 660 epg. The intensity of infection (i.e., geometric mean) by age groups was 74.5 epg (95% CI, 28.4-120.6) in 6–8-year-olds, 89.0 epg (95% CI, 20.9-157.1) in 9-11-year-olds, and 109.9 epg (95% CI, 2.2-217.6) in 12-15-year-olds (*P*=0.246). Higher mean intensity of *Fasciola* species infection was obtained in male children (99.3 epg) compared to females (86.4 epg), though not statistically significant (*P*=0.062).

**Table 3:** Intensity of *S. mansoni* and *Fasciola* species infections by age and gender among the school children in Amhara Regional State, Ethiopia

Characteristics	Intensity of infection^a^ *n* (%)				
	GM (95% CI)	*P* value	1–99 epg	100-399 epg	>400 epg
**Age**					
*S*. *mansoni*					
6-8	86.6 (−34.9-206.9)		21 (65.6)	6 (18.7)	5 (15.6)
9-11	93.2 (51.8-134.6)	0.698^b^	58 (60.4)	31 (32.3)	8 (8.3)
12-15	150.9 (73.9-226.1)		18 (39.1)	21 (45.6)	7 (15.2)
*Fasciola* species					
6-8	74.5 (28.4-120.6)	0.246^b^	4 (80.0)	1 (20.0)	0
9-11	89.0 (20.9-157.1)		10 (55.6)	6 (33.3)	2 (11.1)
12-15	109.9 (2.2-217.6)		6 (46.1)	5 (38.5)	2 (15.4)
**Gender**					
*S*. *mansoni*					
Male	117.3 (57.4-177.2)	0.028^c^	51 (50.0)	37 (36.3)	14 (13.7)
Female	88.2 (38.9-137.5)		45 (62.5)	21 (29.2)	6 (8.3)
*Fasciola* species					
Male	99.3 (13.6-184.9)	0.062^c^	11 (52.4)	7 (33.3)	3 (14.3)
Female	86.4 (17.6-155.2)		9 (60.0)	5 (33.3)	1 (6.7)

Abbreviations: *CI* Confidence interval, *epg* Eggs per gram of stool, *GM* Geometric mean

^a^Determined by Kato-Katz technique

^b^Determined by ANOVA

^c^Determined by Student’s *t* test

### Risk factors for *S*. *mansoni* and *Fasciola* species infections

In the univariate analysis, infection with *S. mansoni* was significantly associated with washing clothes, fetching water, swimming in rivers, bathing in open freshwater bodies, and engaging in irrigation farming. Prevalence of *S. mansoni* infection was significantly different among the six schools, with the highest in the Addis Mender Primary School at 39.7% and the lowest in the Avia Kokit Primary School at 12.8% (*P* < 0.001). The prevalence of *S. mansoni* infection was higher among male children (28.4%) (119/419) compared to females (22.4%) (85/379), but the difference was not statistically significant (Table 1). On the other hand, consuming uncooked vegetables and using unimproved water sources were associated with *Fasciola* species infection. Age, gender, school, irrigation practices, owning domestic animals, and engaging in water contact activities were not associated with *Fasciola* species infection (Table 2).

In the multivariable logistic regression analysis, swimming in rivers (AOR = 1.79; 95% CI, 1.22–2.62; *P* =0.003), bathing in open freshwater bodies (AOR = 2.02; 95% CI, 1.39–2.94; *P* < 0.001), and participating in irrigation farming (AOR = 1.69; 95% CI, 1.19–2.39; *P* = 0.004), attending Addis Mender (AOR = 2.56; 95% CI, 1.20–5.46; *P* =0.015) and Harbu (AOR = 3.53; 95% CI, 1.64–7.59; *P* =0.001) were significantly associated with the prevalence of *S. mansoni* infection, while consuming uncooked vegetables (AOR, 2.47; 95% CI, 1.23-4.97; *P*=0.011) and drinking from unimproved water sources (AOR, 2.28; 95% CI, 1.11–4.70; *P*=0.026) remained significant risk factors for acquiring human fascioliasis (Tables 1 and 2).

## Discussion

Schistosomiasis continues to be a major public health problem in Ethiopia, especially in rural areas where agro-industrial activities are rapidly intensifying and expanding [21, 22]. The prevalence of intestinal schistosomiasis observed in the study area was 25.6 %, in alignment with prevalence of 25.8% in Southern Ethiopia [23], 29.9% in Zegie (Northwest Ethiopia) [24], 24% in Manna District (Southwest Ethiopia) [25], and 29% in Methara (Eastern Ethiopia) [26]. In contrast, the prevalence of *S. mansoni* infection observed in this study was lower than a study conducted in Sanja area, northern Ethiopia (82.8%) [27], Bushulo village, southern Ethiopia (73.7%) [28], and Yachi areas, Southwest Ethiopia (42.9%) [29]. This could be attributed to difference in contamination of water bodies with cercarial stage, the climate of the areas, water contact patterns of the study participants, and socio-economic characteristics.

Our results showed that prevalence and intensity of *S. mansoni* infection were higher among male children than the females. This finding corroborated with other previous studies conducted in South Wello [30], Wondo Genet Zuria [31], Western Abaya [32], Yachi areas [29], and Wolaita Zone [33]. This could be partially explained by the difference in gender-specific water contact activities. In the study area, the male students are likely to engage more in outdoor activities such as playing/swimming in rivers and watering animals while the females are involved more in indoor activities (e.g., cooking and food preparation) [34], which may explain the differences in their exposures to cercariae-infected water.

The prevalence of *S. mansoni* infection was higher in children attending Addis Mender and Harbu Primary Schools. The high prevalence noted in these schools could be related to proximity to nearby water bodies, rendering the children greater opportunities to contaminated water exposure. This is in agreement with findings of earlier studies conducted elsewhere in Ethiopia [29] and Kenya [35], which reported proximity of schools to natural freshwater bodies as an important risk factor for intestinal schistosomiasis. The variation in prevalence between schools could also be related to the difference in other factors, such as fecal contamination of the water bodies and frequency of exposure to open water bodies. Open defecation in field and near water sources is still most commonly practiced in the present study area [13, 36].

The relationship between water contact activities and the occurrence of schistosomiasis is well documented [3, 37]. In our study, the prevalence of *S*. *mansoni* infection was significantly higher among children swimming in rivers and bathing in open freshwater bodies. These water contact activities are a potential risk factor for the children’s exposure to free-swimming cercariae and subsequent infection. This is consistent with the findings of earlier studies that reported a significant correlation between *S. mansoni* infection rate and contact with freshwater bodies [27, 33].

The result of the present study also showed that irrigation practice was significantly associated with *S. mansoni* infection. This observation corroborates previous study which showed that prevalence of *S. mansoni* infection was higher among children who engaged in irrigation activities [11]. Communities in the study area practice small-scale irrigation activities for production of cash crops and cultivation of vegetables. Notably, large water bodies and irrigation canals used for farming and domestic purposes are found in the study area. Such ideal environment will create suitable habitat for the snail intermediate hosts to breed and cercariae to infect the children who often come in contact with contaminated water.

The prevalence of *Fasciola* species infection observed in the present study was 5.5%. This agrees with findings of studies conducted in Lake Tana basin (Northwest Ethiopia) (3.3%) [11] and Kemissie areas (Northeast Ethiopia) (2%) [10]. However, the present study observed a lower prevalence of infection compared to studies conducted in Egypt (12.8%) [38], Vietnam (7.7%) [39], and Peru (9.6%) [40]. These variations could be attributed to differences in ecological variations, dietary habits of the study participants, hygiene, level of environmental contamination, and diagnostic test used. According to the epidemiological classification set for human fascioliasis, areas are considered of hypo-endemic, meso-endemic, and hyper-endemic when they present a prevalence of *Fasciola* species infection below 1%, between 1 and 10%, and greater than 10%, respectively [41]. Hence, the present study area is regarded as meso-endemic region for the disease and this might suggest that the pattern of human fascioliasis distribution is changing from a situation of sporadic human cases to a growing public health problem in the region, highlighting the need for improved surveillance in areas where animal fascioliasis is endemic and irrigation farming are extensively practiced.

The result of the present study shows that there was a significant association between consumption of raw vegetables and *Fasciola* species infection. This result agrees with the study conducted in the Northern Bolivian Altiplano which showed that ingestion of raw aquatic vegetables was a plausible risk factor for the occurrences of human fascioliasis [42]. This could be due to contamination of raw vegetables with metacercariae of *Fasciola* species, whereby the children who frequently eat this kind of food could be infected by these parasites and are able to contaminate the environment through excreting infected feces. Accessing water from unimproved source in the present study was also associated with human *Fasciola* infection. This result is consistent with findings from Egypt [42] and Bolivia [43], which showed that drinking water from unimproved water supplies was linked with human fascioliasis. This could be probably related to the use of contaminated water for drinking and growing vegetables in the area. Lack of improved water supply is the major poverty-related problem reported in the current study area, where majority of the inhabitants use unprotected open water bodies (river, spring, or well) for dinking and recreational purposes, as well as for domestic chores like washing utensils, bathing, and drinking animals. Interventions targeting on the provision of improved water supply and sanitation in the area are needed to reduce the risk of transmission of human fascioliasis and other parasitic infections.

Although the study did not find significant correlation between *S. mansoni* and *Fasciola* infections among the school children across the schools, the spatial distribution of the two diseases shows that five out of six schools were identified as transmission foci of both fascioliasis and schistosomiasis, suggesting a significant overlap of the two parasites which might be of public health implications. This observation is in agreement with earlier studies from Egypt which showed that *Fasciola* species and *S. mansoni* share similar geographic distribution [38, 43]. The overlap in distribution of fascioliasis and schistosomiasis is correlated with the availability of conditions that favor co-existence of both diseases, such as lack of access to safe water supplies, improved hygiene, good sanitation, and existence of favorable environmental conditions for survival of parasites, among others. Given that *S. mansoni* and *Fasciola* species share key similarities in disease transmission and biology, there is a need for further researches that assess the co-morbidities associated with fascioliasis and schistosomiasis co-infection among affected populations. It is also important to highlight the need for integrating surveillance and control of both diseases, especially in areas where they are co-endemic and public health concerns.

Our study has limitations that warrant further studies. First, the data obtained in this study might not be representative of other areas in the country because the distribution of intestinal schistosomiasis and human fascioliasis vary across localities based on ecology and population dynamics of snail hosts. Second, study participants were recruited only from few primary schools. However, human fascioliasis has been shown to cluster within familial groups that share contaminated vegetables or drink from the same water sources [7]. This might have resulted in underestimation of the prevalence of *Fasciola* species infection in the study area. Thus, further population-based surveys involving all age groups are suggested to provide a clearer picture of the diseases in the study area.

## Conclusion

Our study showed that intestinal schistosomiasis and human fascioliasis are prevalent among school children in the study area. Swimming in rivers, bathing in open freshwater bodies, and engaging in irrigation activities were identified as possible risk factors for acquiring intestinal schistosomiasis while consumption of raw vegetables and drinking from unimproved water sources were associated with the prevalence of *Fasciola* species infection. Hence, setting targeted interventions, including administration of anthelmintic drugs, health education, snail control, provision of improved water, and adequate sanitation are needed to control these parasitic infections.

## Data Availability

The data set supporting the conclusions of the study are available from the corresponding author on reasonable request.

## Abbreviations

ALPB: Aklilu lemma institute of pathobiology
AOR: Adjusted odds ratio
CI: Confidence interval
COR: Crude odds ratio
DALYs: Disability-adjusted life years
epg: Egg per gram of stool
IRB: Institutional review board
GM: Geometric mean
LMICs: Low- and middle-income countries
NTD: Neglected tropical disease
SD: Standard deviation
SPSS: Statistical package of social science
WHO: World health Organization
